# Difference in the Electromyographic Behavior of the Masticatory and Swallowing Muscles During Cued Versus Spontaneous Swallowing

**DOI:** 10.1007/s00455-023-10621-x

**Published:** 2023-09-26

**Authors:** Naoya Saito, Toru Ogawa, Naru Shiraishi, Rie Koide, Hideya Komine, Masayoshi Yokoyama, Soshi Hanawa, Keiichi Sasaki

**Affiliations:** Division of Advanced Prosthetic Dentistry, Tohoku University Graduate School of Dentistry, 4-1, Seiryo-machi, Aoba-ku, Sendai, Miyagi 980-8575 Japan

**Keywords:** Electromyography, Mastication, Cued swallowing, Spontaneous swallowing, Dysphagia

## Abstract

The risk of dysphagia and/or aspiration is determined using screening tests, such as the repeated saliva swallowing test and modified water swallowing test, which evaluate cued swallowing. However, humans masticate and swallow foods with various consistencies, forms, and amounts, without conscious awareness. Therefore, this study aimed to examine the difference in the behavior of masticatory and swallowing muscles during spontaneous versus cued swallowing through a series of mastication and swallowing processes by evaluating surface electromyogram (sEMG) signals. The effect of the consistency and amount of food on the behavior of these muscles was also investigated. The sEMG recordings of the masseter muscles and anterior belly of the digastric muscle for 12 subjects, and genioglossus muscle for 5 subjects were obtained. The genioglossus activity was recorded using custom-made ball electrodes. The test foods were cookies and tofu, in amounts of 2 g and 4 g. The normalized muscle activity (integrated EMG), duration of the muscle activity, initial activation timepoint of each muscle, and total duration of swallowing were compared among four conditions. The activity of each muscle was significantly higher during the swallowing of cookies than tofu, for 4 g vs 2 g, and for cued versus spontaneous swallowing. The duration of each muscle activity, initial activation timepoint, and total duration of swallowing were significantly longer for cookies versus tofu, for 4 g vs 2 g, and for spontaneous versus cued swallowing. These results suggest that the behavior of the masticatory and swallowing muscles is affected by cued swallowing and by the consistency and amount of food.

## Introduction

In recent years, the advent of a super-aging society in Japan and an increase in the number of people with dysphagia has led to increased attention on the diagnosis and treatment of dysphagia. It is estimated that about 8 million people have dysphagia [1], which can lead to aspiration pneumonia. Pneumonia is the fifth leading cause of death in Japan, followed by aspiration pneumonia. Approximately 75% of patients hospitalized for pneumonia are older than 70 years. Half of these patients aged 60–70 years were diagnosed with aspiration pneumonia, the incidence of which increases with age [2]. Therefore, it is important to devise a screening test that enables the early and accurate detection of dysphagic symptoms. Ideally, screening tests should be simple, quick, feasible, and minimally invasive, with high sensitivity and specificity [3, 4]. Recently, the Test of Masticating and Swallowing Solids (TOMASS) was developed as a quantitative assessment of solid bolus ingestion, and normative data have been provided, supported by reliability data and validation with instrumental measures [5]. Furthermore, the TOMASS was reported to be a reliable and valid tool in patients with dysphagia, and to distinguish between patients with dysphagia and healthy subjects [6]. The use of the TOMASS in clinical practice may provide a valid and reliable tool for quantitatively measuring the ingestion of solids in patients with dysphagia.

The repeated saliva swallowing test (RSST) and the modified water drinking test (MWST) are currently widely used as screening tests for identifying dysphagia. These screening tests reportedly have high sensitivity and specificity in the detection of dysphagia [7–9]. However, although these tests involve cued swallowing of liquid components (water and saliva), humans usually masticate and swallow foods that have varying consistencies, forms, and amounts, without conscious awareness.

One study investigated whether stage II transport, which is the transfer of the bolus into the oropharynx and accumulation at the epiglottic vallecula during mastication, could be adjusted volitionally in swallowing with mastication [10]. In this previous study, each subject performed two trials with specific instructions. (1) Swallowing without command: the subject ate a cookie in his/her usual manner, and (2) swallowing with command: the subject chewed the cookie, gave a signal when they were ready to swallow, and then swallowed on the command of the investigator. The results showed that volitional swallowing (with command) involved a significantly longer masticatory time and a significantly shorter bolus accumulation time in the oropharynx than swallowing without command. This suggests that in swallowing with mastication, the masticatory movement for the bolus formation, the transfer of the bolus, and the timing of swallowing can be controlled volitionally. However, few detailed studies have considered the influence of volition on the entire process of comprehensive mastication (i.e., from predation to mastication), food transfer from the oral cavity to the pharynx, and swallowing [10, 11].

Differences in the consistency and amount of food also considerably affect masticatory and swallowing behavior [12–14]. The activity time of the suprahyoid muscles becomes longer when swallowing high viscosity food than when swallowing low viscosity food [15], and the number of chewing cycles and the time until swallowing significantly increase when the food is dry [16]. However, although the swallowing cessation duration increases in tandem with the food volume, neither changes in the food viscosity nor changes in taste affect the swallowing cessation duration [17]. Thus, due to interstudy differences in experimental design and limited experimental conditions, there is no consensus on the effects of differences in the consistency and amount of food on the swallowing manner or on cued versus spontaneous swallowing.

In the present study, we examined the difference in the behavior of the masticatory and swallowing muscles (i.e., the masseter muscles, the anterior belly of the digastric muscle, and the genioglossus muscle) during spontaneous versus cued swallowing through a series of mastication and swallowing processes by evaluating surface electromyogram (sEMG) signals. In addition, we investigated the effect of consistency and amount of food on the behavior of these muscles. This study hypothesized that the behavior (i.e., the amount, duration, and timing of activity) of the masticatory and swallowing muscles is different during spontaneous versus cued swallowing (i.e., increased muscle activities, and shorter duration and onset of the activities in cued compared with spontaneous swallowing).

## Methods

### Subjects

The subjects were 12 healthy adults (mean age 29.7 ± 4.2 years; eight men, four women) with no functional abnormality in the maxillofacial and oral regions, and no history of dysphagia. The significance, content, and risks of this study were explained in advance, and all subjects provided written informed consent. To exclude subjects with dysphagia, all subjects underwent the RSST and MWST. The number of participants was determined using the power analysis calculation with the results of pilot experiments. This study was conducted with the approval of the Ethics Committee on Research at the Graduate School of Dentistry, Tohoku University.

### Test Foods

The test foods were cookies (Moonlight Morinaga Seika Co., Ltd.) and tofu (Kokukokutofu Kyuichian Food Cooperative Partnerships), as representatives of solid and semi-solid food, respectively. The IDDSI (International Dysphagia Diet Standardisation Initiative) level of the cookies and tofu was level 7 and level 5, respectively [14]. Each test food was prepared in the amounts of 2 g and 4 g.

### sEMG Recording

**Fig. 1:**
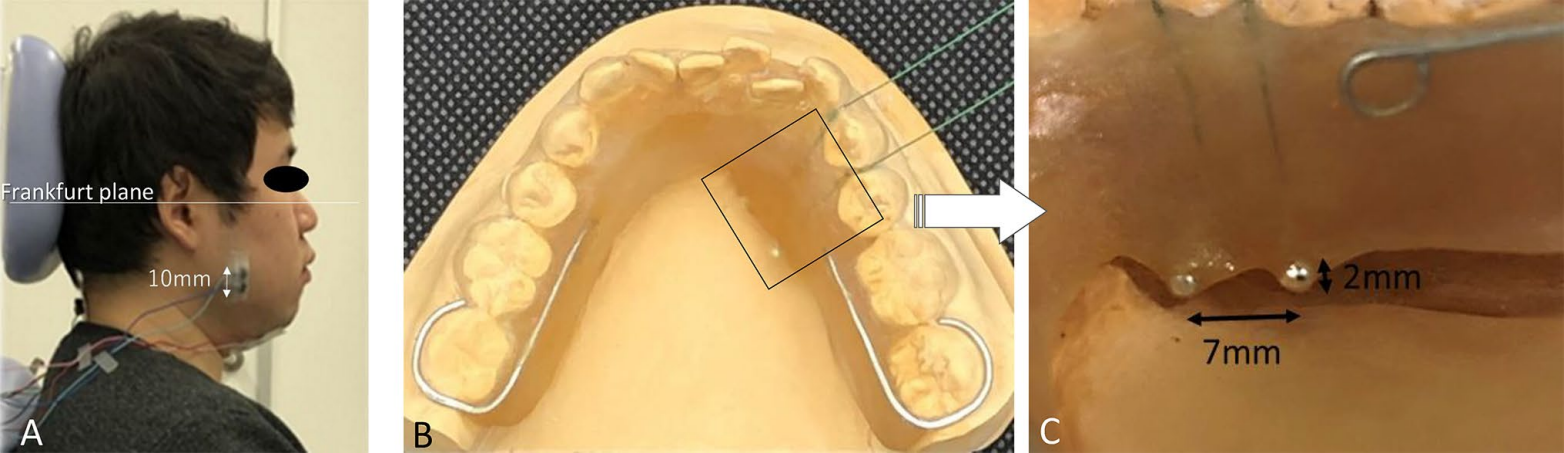
**A** a subject with electrodes placement, **B**, **C** Ball electrodes and electrode-fixing device (experimental splint). The right side is the masticatory side (habitual chewing side) of this subject

Surface EMGs were recorded from the bilateral masseter muscles, the bilateral anterior belly of the digastric muscles, and the genioglossus muscle of the habitual chewing side. The sEMG recordings of the masseter muscles and anterior belly of the digastric muscle were obtained for 12 subjects, and the sEMG of genioglossus muscle was obtained for first 5 consecutive recruited subjects. The habitual chewing side was determined by placing half a piece of cotton roll on the center of the tongue and observing the direction to which the cotton roll was moved during the first chewing cycle [18, 19]. Silver/silver chloride surface disc electrodes (bioelectrode Futami Emu Ih Industries Co., Ltd.) with a diameter of 10-mm were attached to the skin over the center of the masseter muscle and the anterior belly of the digastric muscle. The location of the electrode was determined by palpation of the muscles during tasks involving clenching for the masseter muscles and lifting up the tongue for the anterior belly of the digastric muscle, respectively. The distance between the center of the electrodes was set at 10-mm and the bipolar sEMG signals were derived and recorded. Regarding recording of the anterior belly of the digastric muscle activity, activities of the suprahyoid muscles, such as the geniohyoid and mylohyoid muscles, in the submental region were combined, so that activity of the anterior belly of the digastric muscle was recorded as part of the suprahyoid musculature activities [20, 21]. This compound was also considered with recording of the genioglossus muscle activity. The ground electrode was attached to the back of the neck. The muscle activity of the genioglossus was bipolar derived using custom-made silver ball electrodes (2-mm diameter), which were incorporated in the lingual flange of the experimental splint using a self-curing resin (UNIFAST II-clear, GC). The electrodes were positioned at the border of the lingual flange between the distal right lateral incisor and canine teeth, with a 7-mm distance between electrodes (Fig. 1). A lead wire was passed through the lower interproximal embrasure among the canine tooth and the first and second premolars. Additionally, to enhance the retention of the experimental splint, wire clasps with a diameter of 0.9 mm were attached to the last molars (Fig. 1). The method used to record the activity of the genioglossus muscle was similar to that used in previous studies monitoring the genioglossus activity during respiration [22, 23]. Before the recording sessions, the numbers of masticatory cycles with and without the splint were measured.

### Experimental Procedure

The muscle activity was measured while the subject was sitting on the dental chair in an upright position with the Frankfurt plane parallel to the floor. The subjects could not see the EMG monitor throughout the experiment. Before the recording sessions, the activities of the masseter muscle, anterior belly of the digastric muscle, and genioglossus muscle were recorded during maximum voluntary clenching, tongue thrusting and tongue protrusion to enable the standardized evaluation of sEMG data among subjects. The subjects were asked to take and freely eat a test food (spontaneous swallowing). Then, the mean value X of the number of chewing cycles before swallowing was calculated using the masseter sEMG recordings. For the cued swallowing task, subjects were asked to chew the food X times before swallowing. Recordings of the swallowing of each test food and amount were performed three times in random order. For the sEMG recording of each muscle, the signals were filtered (5 Hz to 1 kHz.) and amplified by a biological amplifier (Multichannel Amplifier MEG-6116 Nihon Kohden Industries Co., Ltd.). After A/D conversion by an A/D converter (Power Lab 16/30 AD Instruments) with a range of 10 V and sampling rate of 2 kHz, the data were stored in a personal computer.

### Data and Statistical Analyses

**Fig. 2:**
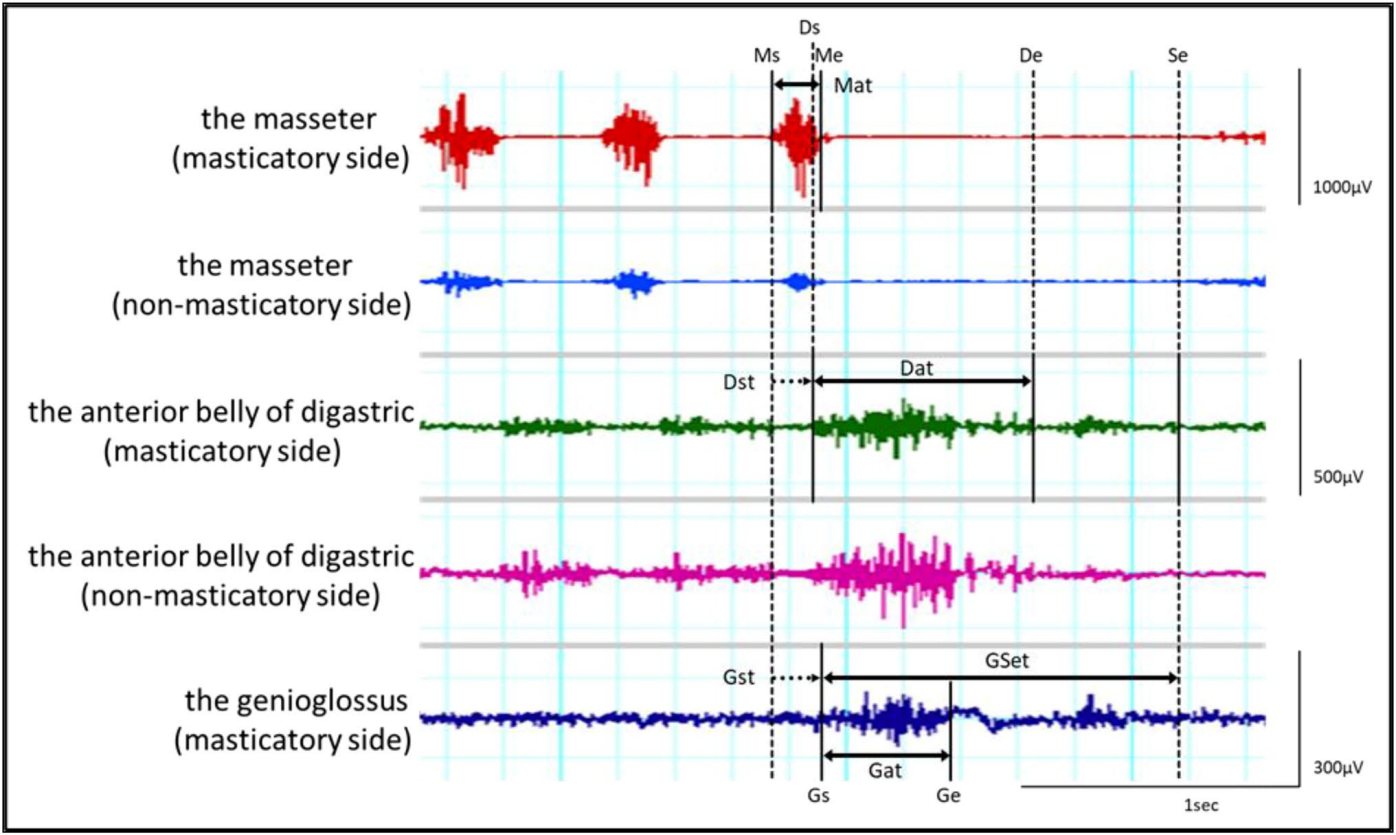
Analyzed parameters. Ms: onset of activity of the masseter muscle, Ds: onset of activity of the anterior belly of the digastric muscle, Gs: onset of activity of the genioglossus muscle, Me: offset of the masseter activity, De: offset of the digastric activity, Ge: offset of the genioglossus activity, Se: offset of swallowing, Mat: duration of the masseter activity, Dat: duration of the digastric activity, Gat: duration of the genioglossus activity, Dst: initial activation timepoint of the anterior belly of the digastric, Gst: initial activation timepoint of the genioglossus, GSet: total duration of swallowing

The obtained muscle activity was analyzed using analysis software (Lab Chart8 AD Instruments), and the outcomes were the sEMG bursts of the masseter muscle, the anterior belly of the digastric muscle and the genioglossus muscle on the habitual masticatory side during chewing and swallowing. The parameters used in the analysis were the normalized sEMG activity (integrated EMG value; iEMG normalized by maximum sEMG values) and duration of the sEMG burst of each muscle, using the onset and offset of the sEMG activity with swallowing. The duration of the sEMG burst was defined as the time from when the amplitude was more than + 3 SD above the resting amplitude to when it was less than + 3 SD below the resting amplitude. The onset of the activity of the anterior belly of the digastric muscle was defined as the onset of swallowing. The initial timepoint of the sEMG bursts of the anterior belly of the digastric muscle and the genioglossus muscle were defined as the time from the offset of the last masseter muscle burst to the onset of activity of each muscle. The end of swallowing was defined when the amplitude of the anterior belly of the digastric muscle become less than + 1 SD of the resting amplitude. The time from the start of the genioglossus muscle activity to the end of swallowing was defined as the total duration of swallowing (Fig. 2). For each subject, the mean value of three trials was used as the representative value for each task, because the Cronbach’s alpha coefficient of the data were more than 0.8.

Statistical analysis was conducted using statistics software (SPSS Statistics v21.0 IBM). Three-factor analysis of variance was performed to evaluate differences between the consistency of the test food (harder cookie or softer tofu), the amount of the test food (2 g or 4 g), and the swallowing style (spontaneous or cued). The significance level was set at P < 0.05.

## Results

All participants passed the swallowing screening procedures. None of the subjects had any swallowing abnormalities detected in the RSST or MWST. There was no difference in the number of chewing movements made with versus without the experimental splint used to fix the electrodes.

**Fig. 3:**
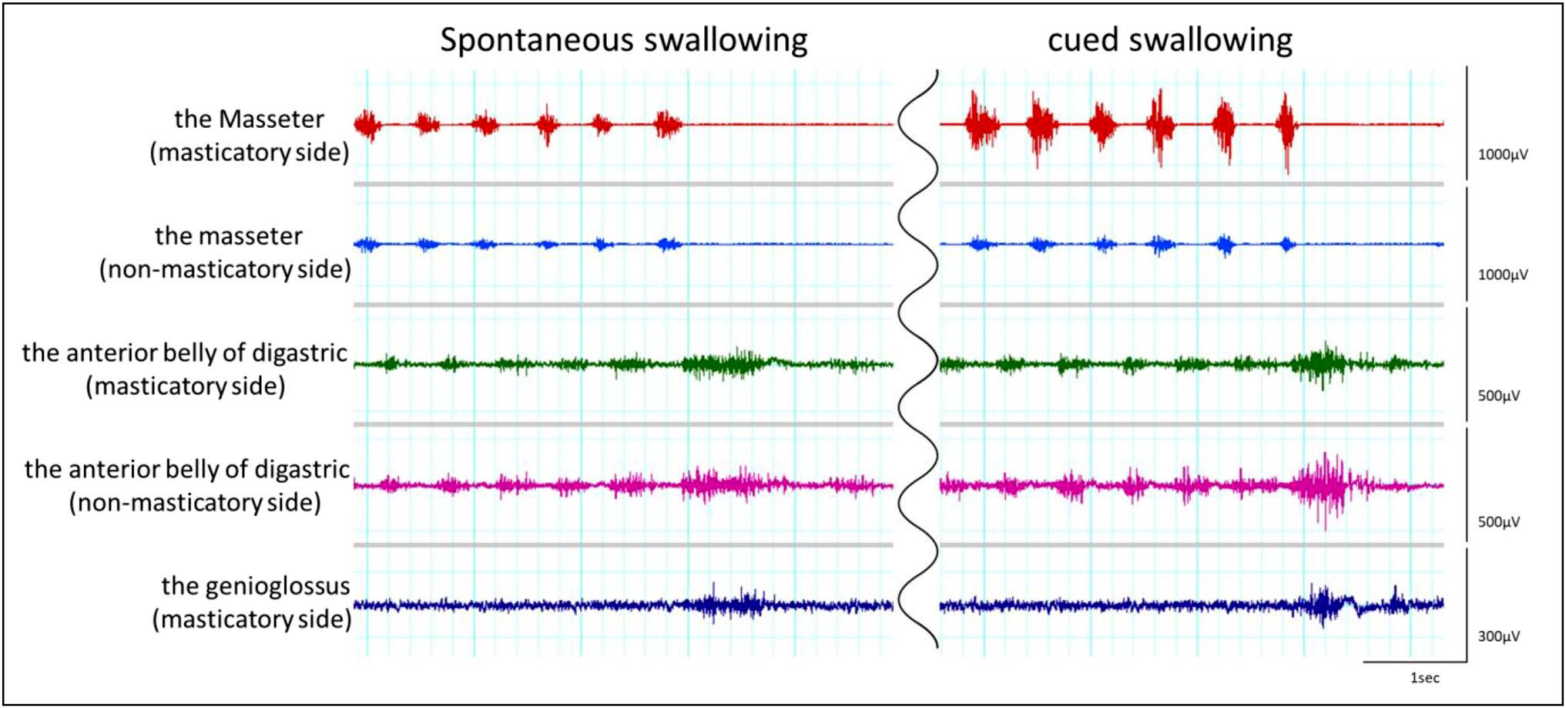
An example of the electromyographic measurements of spontaneous swallowing and cued swallowing when a 4-g cookie is ingested

Figure 3 shows an example of the electromyographic measurement of spontaneous and cued swallowing in an experimental task involving a 4 g cookie. In the original waveforms of all the experimental tasks, after the masseter muscle activities displayed a constant rhythm associated with chewing, the activities of the anterior belly of the digastric muscle and the genioglossus muscle were observed, regardless of the subject, test food, amount of test food, and swallowing style. The anterior belly of the digastric muscle and the genioglossus muscle started their activities almost simultaneously.

**Fig. 4:**
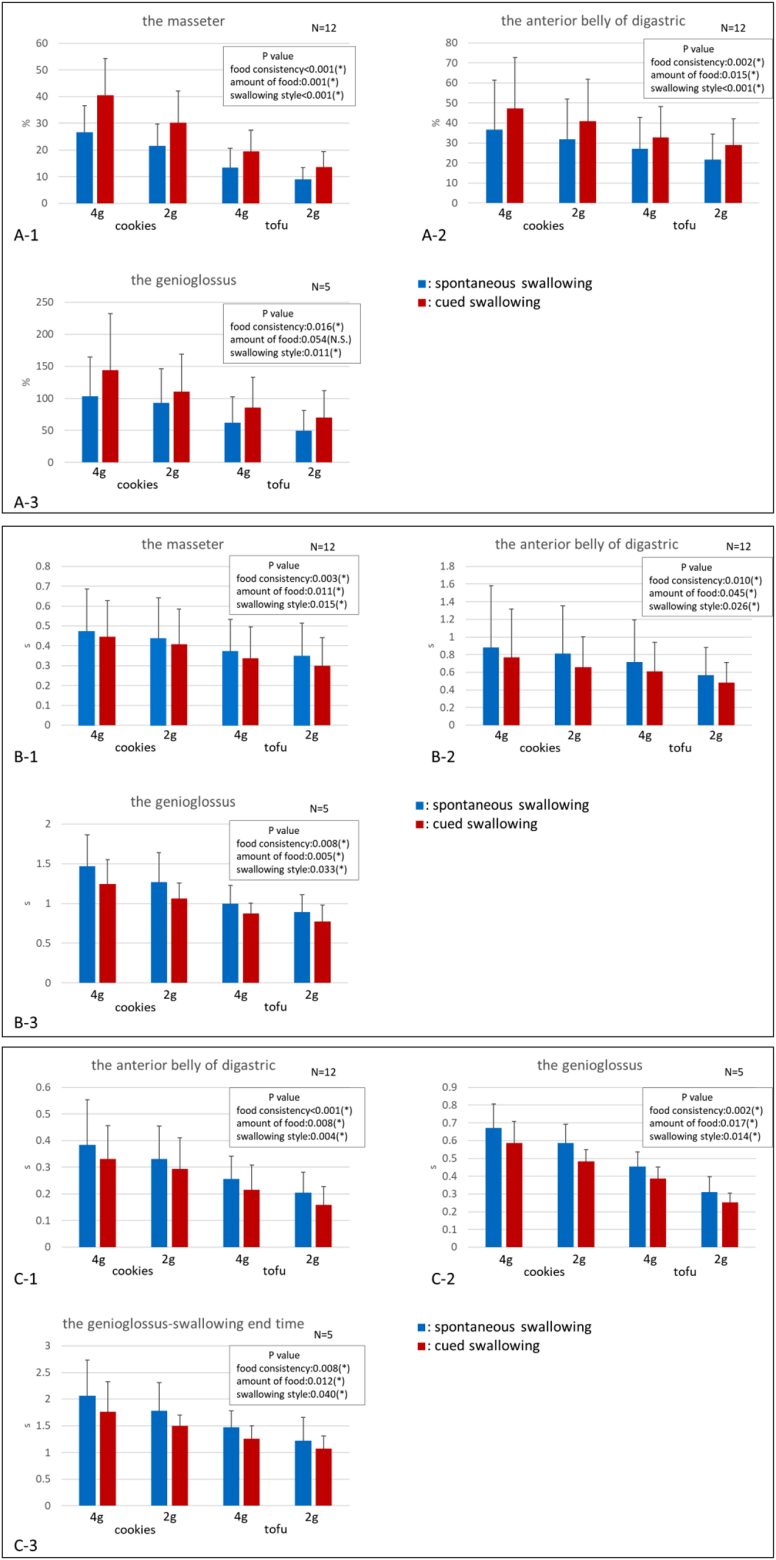
**A**1-3 Graph showing the mean and standard deviation of normalized muscle activity (integrated EMG; iEMG) (%) of the masseter muscle, anterior belly of the digastric muscle, and genioglossus muscle. **B**1-3 Graph showing the mean and standard deviation of the duration of activity of the masseter muscle, anterior belly of the digastric muscle, and genioglossus muscle. **C**1-3 Graph showing the mean and standard deviation of the initial activation timepoint of the anterior belly of the digastric muscle and the genioglossus muscle, and the total duration of swallowing. *Indicates a significant difference, *P* < 0.05. The bar represents the standard deviation

Figure 4 shows the normalized iEMG (A) and duration of activity (B) for each muscle. Figure 4C shows the initial activation timepoint of the anterior belly of the digastric muscle and the genioglossus muscle, and the duration of swallowing from the onset of the genioglossus burst to the end of swallowing.

In the masseter muscles and the anterior belly of the digastric muscle, the iEMG was significantly higher when the subjects were swallowing cookies versus tofu [*P* < 0.001 (masseter), *P* = 0.002 (digastric)], for the 4-g amount of food versus the 2-g amount [*P* = 0.001 (masseter), *P* = 0.015 (digastric)], and in cued swallowing versus spontaneous swallowing (*P* < 0.001 for both the masseter and digastric) (Fig. 4A1–2). The iEMG activity of the genioglossus was significantly increased when the subjects were swallowing cookies versus tofu (*P* = 0.016), and in cued swallowing versus spontaneous swallowing (*P* = 0.011) (Fig. 4A3). There was a marginally significant difference between the 4-g amount of food compared with the 2-g amount (*P* = 0.054). The duration of activity for each muscle was significantly longer when the subjects were swallowing cookies versus tofu [*P* = 0.003 (masseter), *P* = 0.010 (digastric), *P* = 0.008 (genioglossus)], for the 4-g amount of food versus the 2-g amount (*P* = 0.011 (masseter), *P* = 0.045 (digastric), *P* = 0.005 (genioglossus)), and in spontaneous swallowing versus cued swallowing [*P* = 0.015 (masseter), *P* = 0.026 (digastric), *P* = 0.033 (genioglossus)] (Fig. 4B1–3).

The initial activation timepoints of the anterior belly of the digastric muscle and the genioglossus muscle, and the total duration of swallowing were significantly longer when the subjects were swallowing cookies versus tofu [*P* < 0.001 (masseter), *P* = 0.002 (digastric), *P* = 0.008 (genioglossus)], for the 4-g amount of food versus the 2-g amount [*P* = 0.008 (masseter), *P* = 0.017 (digastric), *P* = 0.012 (genioglossus)], and in cued swallowing versus voluntary swallowing [*P* = 0.004 (masseter), *P* = 0.014 (digastric), *P* = 0.040 (genioglossus)] (Fig. 4C1–3). There were no significant interactions among any of the assessed variables (all *P* > 0.05).

## Discussion

Surface electromyography is an effective method of displaying the activities of the masticatory and swallowing muscles during chewing and swallowing [24, 25]. However, as multiple muscles exist in a narrow area in the maxillofacial and oral regions, the activities of the surrounding muscles are combined in the sEMG data of the target muscle [26]. The target muscle activity can be directly derived using needle or wire electrodes, but these electrodes are invasive and cause pain and movement limitation during experimental tasks [27, 28]. As an sEMG recording using a ball electrode is reportedly effective for assessing the muscle activity of the genioglossus [22, 23, 29], we employed this method in the present study. To properly examine the difference between cued and spontaneous swallowing without the effect of the bolus property, we set the same number of chewing cycles in the cued swallowing task and the spontaneous swallowing task. This method was used in a previous study that investigated the effect of attention on chewing and swallowing behaviors by analyzing a fixed number of chewing cycles and fixed chewing duration [11].

To exclude confounding factors such as taste and flavor, the selected test foods were cookies and tofu, which have no taste and flavor types and are considered to be a solid food and a semi-solid food (i.e., IDDSI level 7 and level 5), respectively [14]. Food amounts of 2 g and 4 g were prepared to examine the effect on the muscle activity during swallowing, as amounts of 2 g or 4 g can be safely masticated and swallowed by normal subjects. Regarding the assessed muscles, the masseter was used to estimate the chewing activity. The anterior belly of the digastric muscle was selected as an index of swallowing movement during the pharyngeal phase, as this muscle reportedly shows consistently stable activity during swallowing tasks to elevate the hyoid bone anterosuperior when fixing the mandible [30]. The genioglossus was selected as an index of the tongue motility in the pharyngeal phase of swallowing [31]. The genioglossus protrudes the tongue anteriorly and depresses the middle part of the tongue when the bolus is transported. In addition, the genioglossus pulls the tongue body upward and forward during inspiration and helps prevent the tongue from being drawn into the pharynx and obstructing the airway [22, 23]. It has also been reported that relaxation of the genioglossus and geniohyoid muscles causes obstructive sleep apnea [32]. Thus, genioglossus activity is vital for maintaining patency of the pharynx, especially the oropharynx.

In the present study, the activities of the anterior belly of the digastric muscle and the genioglossus muscle started at almost the same time after the initiation of masseter activity with a constant rhythmical mastication, regardless of the subjects and the experimental tasks. This suggests that after the bolus formation by chewing, the mandible was fixed by the masseter and the other jaw-closing muscles prior to swallowing, and the anterior belly of the digastric muscle and the genioglossus muscle activated cooperatively to elevate the hyoid bone anteriorly and superiorly for swallowing [33–38].

The initial activation timepoint of the anterior belly of the digastric muscle and the genioglossus muscle occurred earlier when the subjects were swallowing tofu versus cookies. One possible reason for this is that the bolus of tofu entered the hypopharynx more quickly than the cookie bolus, so that swallowing was evoked earlier. That is, as the tofu was very soft and mushed very easily and fell into the pharynx at an early stage, this caused the early induction of the swallowing reflex with the activity of the anterior belly of the digastric muscle and the genioglossus muscle. It has been reported that swallowing after chewing does not change the amount of forward movement of the hyoid bone, but increases the amount of upward elevation compared with swallowing liquid without chewing [39]. This is consistent with the present findings that swallowing the harder cookie resulted in a longer duration of activity of the genioglossus muscle, a longer total duration of swallowing, and an increase in sEMG activity compared with swallowing the tofu, as the genioglossus muscle can protrude the tongue and hyoid bone anteriorly and elevate these structures superiorly. This might be attributed to temporal and structural coordination depending on the bolus properties in the pharyngeal region [37].

The muscle activity was decreased when the subjects were swallowing 2 g of food vs 4 g of food. This may be because of the reduction in the load amount due to the decrease in the size of the bolus formed by chewing. In addition, the masticatory time was shortened during swallowing of the smaller food amount, so that it flowed into the pharynx earlier; this may be the reason for the earlier initial activation timepoint of the anterior belly of the digastric muscle and the genioglossus muscle, and the shorter total duration of swallowing for the 2 g amount versus the 4 g amount.

In spontaneous swallowing with chewing, the bolus is sequentially sent to the pharynx (even during mastication) and accumulates in the pharynx [10]. Accordingly, this is the reason for the prolonged duration of activity of the anterior belly of the digastric muscle and the genioglossus muscle during spontaneous versus cued swallowing. In contrast, in cued swallowing, the bolus is retained in the oral cavity until swallowed due to an awareness of chewing. Therefore, the activities of the masseter muscle, anterior belly of the digastric muscle, and genioglossus muscle increased as the total amount of bolus was transferred to the oropharynx during swallowing. However, if sufficient bolus formation cannot be achieved due to a masticatory disorder, the encouragement of cued chewing and swallowing may increase the risk of aspiration due to the malformed bolus remaining in the epiglottic vallecula after swallowing. In addition, excessive attention to masticatory movements may alter the cooperative relationship between the masticatory and swallowing functions; further studies are needed to investigate these effects.

The swallowing reflex is induced when the stimulus intensity reaches a certain threshold level. The swallowing reflex is not triggered by a weak stimulus that does not reach the threshold level, while the choke reflex occurs if it is judged to be an inappropriate signal due to an unsuitable bolus for swallowing. This feedback adjustment in the swallowing reflex mechanism means that the risk of aspiration must be increased when the swallowing reflex is evoked even the presence of unsuitable food for swallowing in the oral cavity during chewing. Furthermore, spontaneous swallowing with chewing, which sends the appropriate bolus for swallowing to the pharynx (even during mastication) to accumulate in the pharynx, may be induced in accordance with the airway defenses for safe respiration, i.e., feed-forward adjustment. When liquid is directly injected into the pharynx using a catheter, an airway-defensive swallowing reflex against nociception and aspiration is evoked [40]. The spontaneous swallowing reflex with chewing might be a similar reflexive phenomenon to prevent aspiration. Therefore, spontaneous swallowing has an airway-defensive function and improves the efficiency of chewing while sequentially transferring the masticated bolus to the pharynx.

The present results confirmed that surface sEMG signals assisted in evaluating the swallowing function. To develop an effective swallowing screening test and construct a swallowing training method, it might be necessary to consider the swallowing styles (cued and spontaneous). The goal of swallowing training is to establish a method that enables safe swallowing without aspiration. However, this is not easy to achieve because the respiratory and food pathways are open at the same time during the pharyngeal phase [37, 41]. In addition, a bolus may reach the hypopharynx during chewing due to the influence of gravity and may induce the swallowing reflex at an inappropriate time, resulting in an increased risk of aspiration [42]. Hence, when feeding food in swallowing training, it is important to comprehensively consider the physical characteristics of the food, the effects of gravity, and the swallowing style. In this study, differences in muscle activities were found between spontaneous versus cued swallowing, suggesting the importance of providing instructions for mastication and swallowing during eating training, research tasks, and screening tests for dysphagia.

The present study examined the activities of the masticatory and swallowing muscles using surface sEMG during a series of chewing and swallowing functions. However, the study has several limitations. As the hyoid bone or epiglottis were not monitored (i.e., were not simultaneously assessed using videofluorographic or videoendoscopic evaluation), the onset of suprahyoid muscle (digastric) activity was defined as the onset of swallowing, while the timepoint at which the suprahyoid muscle (digastric) relaxed and the hyoid bone returned to its original position was defined as the end of swallowing. Although several previous studies have also used sEMG recordings to evaluate the anterior belly of the digastric muscle and the genioglossus muscle [22, 23, 32–34], it is possible that the sEMG signals of the target muscles were mixed with signals from adjacent muscles (e.g., the mylohyoid and geniohyoid muscles in the anterior belly of the digastric muscle, and the mylohyoid muscle in the genioglossus muscle).

## Conclusion

Regardless of changes in the consistency and amount of food, compared with voluntary swallowing, cued swallowing increased the activity of the masticatory and swallowing muscles, extended the duration of the muscle activity, and hastened the initial activation timepoints of the muscles. This suggests that cued swallowing, food consistency, and amount of food may affect the behavior of the masticatory and swallowing muscles.
